# A Comprehensive Literature Review of the CDH1 Mutation and Its Role in Gastric Cancer

**DOI:** 10.7759/cureus.85072

**Published:** 2025-05-30

**Authors:** Malik Samardali, Jehad Samardaly, Ibrahim Shanti

**Affiliations:** 1 Internal Medicine, St. Elizabeth Youngstown Hospital - NEOMED (Northeast Ohio Medical University) Program, Youngstown, USA; 2 Internal Medicine, Jordan University of Science and Technology, Ajloun, JOR; 3 Internal Medicine, Joan C. Edwards School of Medicine - Marshall University, Huntington, USA

**Keywords:** cdh1 mutation, e-cadherin, gastric cancer, hereditary diffuse gastric cancer, prophylactic gastrectomy

## Abstract

Hereditary diffuse gastric cancer (HDGC) is a significant genetic predisposition syndrome, primarily caused by germline mutations in the CDH1 gene, which encodes the E-cadherin protein - a critical component for cell adhesion and tissue integrity. Individuals with HDGC have an increased risk of developing diffuse-type gastric cancer, which is characterized by the infiltration of the stomach wall by poorly cohesive, single-cell carcinoma. This aggressive form of gastric cancer is challenging to detect at an early stage and is associated with a poor prognosis. The identification and understanding of the genetic basis of HDGC are crucial for improving early detection, risk assessment, and the development of targeted treatment strategies for this hereditary cancer syndrome. This review aims to provide a comprehensive overview of the genetic and molecular mechanisms underlying HDGC, with a focus on the role of CDH1 mutations. It discusses current diagnostic criteria, genetic testing strategies, and the implications of a positive CDH1 mutation for clinical management - including surveillance, prophylactic gastrectomy, and family counseling. Additionally, this review highlights recent advancements in understanding the impact of CDH1 loss on cellular pathways and the potential for novel therapeutic approaches targeting these molecular changes. By consolidating the existing knowledge and addressing current gaps, this review aims to support clinicians and researchers in enhancing the care and outcomes of individuals and families affected by HDGC.

## Introduction and background

Gastric cancer is a significant global health concern, recognized as one of the most prevalent malignancies and a leading cause of cancer-related mortality. It ranks as the fifth most common cancer and the third leading cause of cancer deaths worldwide, with an estimated 1 million new cases diagnosed annually [1]. The disease is particularly prevalent in East Asia, Eastern Europe, and parts of South America, where environmental factors, dietary habits, and genetic predispositions contribute to its incidence [1,2]. The high mortality rate associated with gastric cancer is largely attributed to late-stage diagnosis and the aggressive nature of the disease [3].

Gastric cancer is characterized by its heterogeneity, which is evident in its various subtypes. The Cancer Genome Atlas (TCGA) project has classified gastric cancer into four distinct molecular subtypes based on genetic and epigenetic alterations: Epstein-Barr virus (EBV)-positive, microsatellite instability (MSI), genomically stable (GS), and chromosomal instability (CIN) [4-6]. Each subtype exhibits unique biological behaviors and clinical outcomes, influencing treatment strategies and prognostic assessments. For instance, the EBV-positive subtype is associated with specific mutations and a distinct immune profile, while the MSI subtype is characterized by high levels of MSI and often responds well to immunotherapy [5,6]. The GS subtype, on the other hand, is linked to epithelial-to-mesenchymal transition (EMT) features, suggesting a more aggressive clinical course [4]. In addition to these molecular classifications, gastric cancer can also be categorized based on histological features. The two primary histological types, as per Lauren's classification, are intestinal and diffuse gastric cancer (DGC). Intestinal-type gastric cancer is often associated with environmental factors and is characterized by glandular structures, while diffuse-type gastric cancer is more aggressive and lacks distinct glandular formation [7-9]. This distinction is crucial, as it influences both the clinical management and the prognosis of the disease.

The pathogenesis of gastric cancer is multifactorial, with *Helicobacter pylori* infection being a significant risk factor. Chronic infection with *H. pylori* is linked to the development of gastric atrophy and intestinal metaplasia, which are precursors to gastric cancer [10]. Other contributing factors include genetic predispositions, dietary influences, and lifestyle choices, which collectively underscore the complexity of gastric cancer as a public health issue [1,2]. Recent studies have also highlighted the role of various biomarkers and genetic alterations in gastric cancer prognosis. For example, the expression of specific RNA-binding proteins has been linked to survival outcomes, suggesting potential avenues for personalized treatment strategies [11]. Furthermore, the identification of CIN as a hallmark of certain gastric cancer subtypes has opened new research pathways aimed at understanding the molecular mechanisms driving tumorigenesis [12].

Among the genetic factors that influence gastric cancer, hereditary diffuse gastric cancer (HDGC) is a significant hereditary syndrome characterized by a high risk of developing gastric cancer, primarily due to mutations in the CDH1 gene, which encodes E-cadherin. This autosomal dominant condition accounts for approximately 1%-3% of all gastric cancer cases. The CDH1 gene is located on chromosome 16, and its mutations lead to a loss of cell adhesion, promoting the invasive characteristics of gastric cancer cells [13]. Recent studies have highlighted the role of various genetic variants beyond CDH1 in gastric cancer susceptibility. For instance, Zhao et al. identified multiple genetic loci associated with gastric cancer risk through genome-wide association studies (GWAS), emphasizing the multifactorial nature of the disease [14]. Furthermore, genetic variations in genes such as XRCC1 and ERCC5 have been linked to increased susceptibility to gastric cancer, indicating that DNA repair mechanisms may play a critical role in the disease's etiology [15,16]. These findings suggest that while CDH1 mutations are pivotal in HDGC, other genetic factors contribute to the overall risk of gastric cancer.

The interaction between genetic predispositions and environmental factors is also crucial in understanding gastric cancer development. For example, the presence of *H. pylori* infection, a well-established risk factor for gastric cancer, may interact with genetic susceptibility to exacerbate cancer risk [17]. Genetic variants in inflammatory mediator genes, such as those encoding components of the NF-κB signaling pathway, have been associated with increased gastric cancer risk, further illustrating the interplay between genetic predisposition and environmental influences [18]. In addition to CDH1, other genes have been implicated in familial gastric cancer syndromes. For instance, mutations in the ATM gene have been associated with a moderately increased risk for gastric cancer, although its role is less understood compared to CDH1 [19]. Moreover, research has identified additional genetic variants, such as those in MAP3K6, that may confer risk for familial gastric cancer, suggesting that multiple genetic pathways may be involved in the disease's familial forms [20]. Therefore, the role of genetics in gastric cancer, particularly in HDGC, is multifaceted. While CDH1 mutations are central to HDGC, a broader spectrum of genetic variations contributes to gastric cancer susceptibility.

The CDH1 gene, which encodes the protein E-cadherin, plays a critical role in cell adhesion and maintaining epithelial integrity. Mutations in this gene are primarily associated with HDGC, an autosomal dominant cancer predisposition syndrome. HDGC is characterized by a high lifetime risk of developing gastric cancer - estimated at 70% for men and 56% for women by age 80 for mutation carriers - alongside a significant risk for lobular breast cancer (LBC) in female carriers, which is approximately 42% by age 80 [21,22]. The identification of germline mutations in CDH1 has transformed the understanding of familial gastric cancer syndromes, highlighting the importance of genetic screening in at-risk populations [23].

The significance of the CDH1 gene in cancer, particularly in the context of HDGC, lies in its role as a tumor suppressor. E-cadherin is crucial for maintaining the structural integrity of epithelial tissues, and its loss or dysfunction can lead to increased cellular motility and invasiveness - hallmarks of cancer progression [24]. In HDGC, the inactivation of the second allele of the CDH1 gene often occurs through somatic mutations or hypermethylation, consistent with the "two-hit" hypothesis of tumorigenesis [25,26]. This loss of E-cadherin expression is a hallmark of DGC, resulting in a distinct histological pattern characterized by poorly cohesive signet-ring cell carcinoma (SRCC) [25]. Studies have shown that somatic mutations and epigenetic alterations, such as promoter methylation of CDH1, are prevalent in sporadic cases of gastric cancer, particularly in the diffuse type, where they are associated with poor clinical outcomes [27,28]. This underscores the dual role of CDH1 mutations in both hereditary and sporadic gastric cancer contexts.

Furthermore, the clinical implications of CDH1 mutations extend beyond gastric cancer. Research indicates that individuals with CDH1 mutations may benefit from proactive surveillance strategies, including endoscopic screening and prophylactic surgeries, to mitigate cancer risk [29]. The identification of CDH1 mutations in families with a history of gastric cancer has led to the establishment of specific testing criteria, enabling targeted genetic counseling and management strategies [23]. Understanding the genetic underpinnings of HDGC not only aids in risk assessment and management of affected families but also contributes to the broader understanding of gastric cancer biology. The significance of CDH1 mutations extends beyond individual cases, influencing clinical guidelines and preventive measures for hereditary cancer syndromes.

## Review

Biology of the CDH1 mutation

CDH1 Gene Function

The CDH1 gene, located on chromosome 16q22.1, encodes the protein E-cadherin, which is a critical component of adherens junctions in epithelial tissues. E-cadherin functions primarily as a calcium-dependent cell adhesion molecule that facilitates cell-cell adhesion, thereby maintaining epithelial integrity and polarity. This adhesion is essential for the structural organization of tissues and plays a significant role in various biological processes, including embryogenesis and tissue homeostasis [30].

E-cadherin's role extends beyond mere adhesion; it is also implicated in the regulation of cellular signaling pathways that influence cell proliferation and differentiation. Loss of E-cadherin function is frequently associated with EMT, a process that enables cancer cells to acquire migratory and invasive properties, facilitating metastasis. For instance, studies have shown that downregulation of E-cadherin correlates with increased cell motility and a higher likelihood of cancer progression in various malignancies, including ovarian and gastric cancers [28]. The loss of E-cadherin expression has been documented in numerous cancer types, indicating its potential role as a tumor suppressor [31].

Moreover, the regulation of E-cadherin expression is influenced by epigenetic modifications, such as promoter methylation, which have been observed in several cancers, including cervical and bladder cancers. The methylation of the CDH1 promoter can lead to decreased E-cadherin expression, further contributing to the invasive characteristics of tumors [32]. Additionally, genetic mutations in the CDH1 gene are linked to HDGC, underscoring its importance in cancer predisposition [31,33]. Individuals with pathogenic germline mutations in CDH1 face a significantly elevated risk of developing gastric and LBCs [33].

Mechanism of Mutation

Various types of mutations in the CDH1 gene can lead to the disruption of its protein function, contributing to cancer susceptibility. The most common types of mutations include missense, nonsense, frameshift, and splice-site mutations, each affecting the protein in different ways.

The missense mutations result in a single amino acid change in the protein sequence. They are the most frequently observed mutations in the CDH1 gene, accounting for approximately 28% of all identified alterations [34]. Some missense mutations can destabilize the E-cadherin protein, leading to misfolding and subsequent degradation via the endoplasmic reticulum-associated degradation (ERAD) pathway [34]. For instance, the missense variant c.1679C>G (p.T560R) has been shown to disrupt normal splicing, further complicating its functional impact [35,36]. Such mutations can impair the adhesive properties of E-cadherin, which is critical for maintaining epithelial integrity, thus promoting tumorigenesis [37]. The nonsense mutations introduce a premature stop codon in the coding sequence, resulting in truncated proteins that are typically nonfunctional. Nonsense mutations are less common but are significant because they lead to a complete loss of E-cadherin function, which is essential for cell adhesion [34]. The absence of functional E-cadherin can facilitate the invasive characteristics of cancer cells, contributing to the aggressive nature of DGCs associated with CDH1 mutations [38]. The frameshift mutations occur due to insertions or deletions of nucleotides that alter the reading frame of the gene. Frameshift mutations can lead to the production of entirely aberrant proteins or truncated versions, similar to nonsense mutations. They are responsible for a significant proportion of CDH1 mutations, particularly in familial gastric cancer cases [34]. The resultant proteins often lack critical functional domains necessary for E-cadherin's role in cell adhesion, further promoting cancer progression. The splice-site mutations affect the splicing of pre-mRNA, potentially leading to the exclusion of essential exons or the inclusion of introns in the mature mRNA. Such alterations can result in dysfunctional proteins that may not be able to perform their normal cellular functions [34]. For example, splice-site mutations can lead to the production of E-cadherin variants that are unstable or unable to localize properly to the cell membrane, thus impairing cell adhesion and promoting tumorigenic processes [39].

Other mutations include loss of heterozygosity (LOH), promoter region hypermethylation, and copy number variations (CNVs). LOH occurs when one allele of the CDH1 gene is lost in a tumor cell, resulting in the loss of functional E-cadherin. LOH is a common event in many cancers and can lead to a complete loss of E-cadherin expression if the remaining allele is also mutated or silenced. The promoter region of the CDH1 gene can undergo hypermethylation, which silences gene expression without altering the DNA sequence [40-42]. This epigenetic modification can lead to reduced or absent E-cadherin protein levels, contributing to tumor invasiveness. CNVs involve changes in the number of copies of the CDH1 gene, which can result in either gene amplification or deletion. CNVs can affect the overall expression levels of E-cadherin [41].

HDGC syndrome

Overview of HDGC

HDGC is a clinically significant autosomal dominant syndrome, primarily associated with germline mutations in the CDH1 gene, which encodes the E-cadherin protein. This condition is characterized by a markedly increased risk of developing DGC and LBC, with lifetime risks estimated at 42%-70% for males and 33%-56% for females carrying pathogenic CDH1 mutations [43,44]. The clinical manifestation of HDGC is often insidious, with many patients presenting with advanced disease due to the lack of early symptoms. The International Gastric Cancer Linkage Consortium (IGCLC) has established clinical criteria for identifying families at risk, emphasizing the importance of genetic testing for CDH1 mutations in individuals with a family history of gastric or LBC [28]. Studies have shown that approximately 25%-30% of families meeting these criteria harbor germline alterations in the CDH1 gene, underscoring the need for systematic genetic screening in at-risk populations [45].

The penetrance of CDH1 mutations in families with HDGC is significant, with studies suggesting that 70%-80% of carriers may develop associated cancers [46]. However, not all identified CDH1 mutations are pathogenic, as some may not meet the criteria for HDGC, highlighting the complexity of genetic counseling and risk assessment in these families [47]. The clinical implications of HDGC necessitate proactive management strategies, including regular surveillance and consideration of prophylactic measures. Prophylactic total gastrectomy (PTG) is often recommended for individuals with confirmed CDH1 mutations, particularly given the tendency for gastric cancer to present at an advanced stage with minimal prior symptoms. This approach aims to mitigate the high cancer risk associated with the syndrome, although it is accompanied by significant surgical risks and long-term implications for quality of life (QoL). Additionally, enhanced breast cancer screening, including annual mammograms and MRI, is advised for women with CDH1 mutations, starting at age 35, reflecting the increased risk of LBC in this population [48]. Thus, the identification of CDH1 mutations not only aids in cancer risk stratification but also informs clinical decision-making regarding surveillance and preventive strategies.

Diagnostic Criteria

HDGC is a genetic syndrome first described by the IGCLC in 1999. It is characterized by the presence of either (1) two or more confirmed cases of DGC in first- or second-degree relatives, with at least one diagnosed before 50 years of age; or (2) three or more cases of DGC in first- or second-degree relatives, regardless of the age at diagnosis [49].

The clinical criteria for diagnosing HDGC primarily focus on families with a history of DGC or LBC. The diagnosis is considered when a family has two or more cases of DGC among first- or second-degree relatives, with at least one diagnosed before the age of 50. Additionally, HDGC can be diagnosed if there is at least one case of LBC in a first- or second-degree relative, especially when accompanied by a family history of DGC. The identification of a pathogenic germline mutation in the CDH1 gene in an affected individual or family member further supports an HDGC diagnosis. Even if families do not meet the established criteria, multiple cases of DGC or LBC within a family may still warrant consideration for HDGC evaluation [50]. These criteria are designed to aid in identifying individuals and families at risk for HDGC, facilitating appropriate genetic counseling, testing, and management strategies.

PTG as a preventive strategy

Rationale for Prophylactic Surgery

PTG is recommended for individuals with CDH1 mutations, primarily due to the significantly elevated risk of developing DGC associated with these mutations. CDH1, which encodes the E-cadherin protein, is a critical tumor suppressor gene, and mutations in this gene lead to a high penetrance of gastric cancer, with estimates indicating a cumulative risk of 40%-67% for men and 63%-83% for women by age 75 [31]. The mean age of diagnosis for gastric cancer in CDH1 mutation carriers is approximately 38 years, and the presence of multiple microscopic foci of cancer is common, even in asymptomatic individuals [51,52].

The rationale for recommending PTG stems from the fact that current surveillance methods, such as endoscopy, are inadequate for early detection of gastric cancer in these high-risk individuals. Studies have shown that up to 92% of patients undergoing prophylactic gastrectomy had undetected SRCC foci in their resected specimens, indicating that the cancer can be present without any overt clinical symptoms [53]. Furthermore, the high mortality associated with advanced gastric cancer underscores the necessity of preventive measures, as, once diagnosed, the prognosis is often poor due to late-stage presentation [52].

In addition to the gastric cancer risk, individuals with CDH1 mutations also face a heightened risk of LBC, with estimates ranging from 39% to 52% [31]. This dual risk further complicates the management of individuals with CDH1 mutations, making prophylactic surgery a critical intervention. PTG not only reduces the risk of gastric cancer but also allows for better management of breast cancer risk through enhanced surveillance strategies [29]. Overall, the recommendation for PTG in CDH1 mutation carriers is supported by the high penetrance of gastric cancer, the limitations of current surveillance techniques, and the potential for improved outcomes through early intervention [34].

Timing of Surgery

The timing of this surgical intervention is typically recommended in early adulthood, particularly between the ages of 18 and 40, due to the significant lifetime risk of developing DGC, which can exceed 80% by age 80 for mutation carriers [48]. The rationale for early intervention is based on the aggressive nature of HDGC, which often presents with advanced disease at diagnosis, leading to poor prognoses if surgery is delayed until symptoms arise [54].

Several factors influence the decision regarding the timing of surgery. One of the primary considerations is the patient's age and reproductive plans. For younger women, the metabolic consequences of total gastrectomy during pregnancy can be a significant concern, leading some experts to suggest that a delay in surgery may be reasonable until after childbearing is completed. However, recent studies indicate that successful pregnancies can occur post-gastrectomy, which may alleviate some concerns [34]. Additionally, the psychological impact of undergoing such a significant surgical procedure at a young age can also weigh heavily on decision-making, as patients may grapple with the implications for their QoL and nutritional status post-surgery [55].

Moreover, the presence of family history and personal medical history plays a crucial role in determining the timing of PTG. Individuals with a strong family history of gastric cancer or those who have experienced early-onset breast cancer may be more inclined to pursue early surgical intervention [31]. Genetic counseling is essential in this context, as it helps patients understand their risks and the potential benefits of prophylactic surgery, thereby facilitating informed decision-making [56]. Furthermore, the effectiveness of regular surveillance methods, such as endoscopy, has been called into question, with evidence suggesting that these approaches may not adequately detect early-stage cancers in mutation carriers [31,56].

Thus, the decision to undergo PTG for individuals with CDH1 mutations is multifaceted, involving considerations of age, reproductive plans, family history, psychological readiness, and the limitations of surveillance strategies. The consensus among experts is to recommend PTG in early adulthood to mitigate the high risk of developing DGC, while also recognizing the need for personalized approaches based on individual circumstances and preferences [55].

Surgical Outcomes

The decision to undergo PTG is influenced by various factors, including the potential benefits, risks, and outcomes associated with the procedure. The primary benefit of PTG is the significant reduction in the risk of developing gastric cancer. Individuals with CDH1 mutations face a cumulative risk of gastric cancer that can reach up to 83% by age 80, with women having a slightly higher risk compared to men [33,37]. Prophylactic gastrectomy is currently the only reliable preventive measure for these individuals, effectively eliminating the risk of gastric cancer [54]. Furthermore, the aggressive nature of HDGC necessitates early intervention, as symptomatic cases often present at advanced stages, leading to poor prognoses.

However, the procedure is not without its risks and complications. Research has shown that patients undergoing PTG may experience significant postoperative challenges, including nutritional deficiencies, changes in body image, and psychological impacts such as anxiety and depression [57,58]. A systematic review highlighted that health-related QoL often declines immediately following surgery but tends to improve within 6 to 12 months, although some patients may continue to experience long-term effects on their QoL [34,58]. Additionally, cognitive recovery can be slow, with some patients reporting difficulties in eating and adjusting to their new lifestyle post-surgery [54].

The surgical technique employed can also influence outcomes. Laparoscopic total gastrectomy has been shown to result in fewer complications, reduced blood loss, and shorter hospital stays compared to open surgery, making it a preferable option for prophylactic procedures [59]. However, the decision to proceed with PTG should be made with careful consideration of the individual’s health status, family history, and personal preferences, as the procedure carries inherent risks of morbidity [60]. Moreover, genetic counseling plays a crucial role in the decision-making process. Individuals with CDH1 mutations often face complex emotional and psychological challenges, and understanding the implications of their genetic status can help guide their choices regarding prophylactic surgery. The need for comprehensive support systems is emphasized, as patients may have unique informational needs and varying levels of perceived risk associated with gastric cancer [61].

QoL Post-surgery

The QoL for individuals with CDH1 mutations who undergo PTG to prevent DGC is a complex issue, influenced by various factors, including digestive and nutritional challenges. Studies have shown that, while PTG significantly reduces the risk of developing gastric cancer, it also leads to substantial changes in patients' QoL, particularly in the immediate postoperative period. One of the primary findings regarding QoL post-gastrectomy is that patients often experience a decline in health-related QoL immediately following the surgery. For instance, a study involving 32 patients indicated that health-related QoL scores dropped significantly after total gastrectomy, with improvements noted at 6-12 months postoperatively, although some decline was observed again at 24 months [54]. This initial decline can be attributed to the physical and psychological adjustments required after such a major surgical intervention.

Nutritional challenges are a significant concern for patients post-gastrectomy. The absence of the stomach leads to difficulties in food retention and digestion, which can result in malnutrition and weight loss. Research indicates that patients typically experience weight loss of about 10%-20% after surgery, primarily due to the reduced capacity for food intake and altered digestion processes - often referred to as post-gastrectomy syndrome [62]. This syndrome encompasses a range of symptoms, including early satiety, dumping syndrome, and nutritional deficiencies, which can further impact QoL [61]. Moreover, the psychological impact of living without a stomach can lead to issues such as body image dissatisfaction and anxiety about eating. In a study, 44% of patients reported a poor body image following surgery, highlighting the emotional toll of the procedure [61]. Additionally, persistent gastrointestinal symptoms such as diarrhea, reflux, and strict dietary restrictions can continue to affect patients long after the initial recovery period, complicating their ability to return to normal life [61].

To mitigate these challenges, nutritional interventions are critical. Some studies have shown that early nutritional support, such as the use of elemental diets, can help manage weight loss and improve overall nutritional status in the postoperative period. Furthermore, ongoing dietary counseling and support can assist patients in adapting to their new eating habits and in managing the psychological aspects of their condition [61].

Surveillance and management for non-surgical patients

Endoscopic Surveillance

The management of individuals with CDH1 mutations, particularly those who choose to delay or decline prophylactic surgery, necessitates a robust endoscopic surveillance strategy. CDH1 mutations are associated with HDGC, which carries a high risk of developing gastric cancer, with penetrance estimates ranging from 50% to 80%, depending on various factors such as gender and ethnicity [43,63]. Given this significant risk, the IGCLC recommends PTG for individuals with CDH1 mutations, ideally between the ages of 20 and 30 [43]. However, for those who opt against surgery, intensive endoscopic surveillance becomes crucial.

Endoscopic surveillance protocols have been developed specifically for individuals with CDH1 mutations who decline prophylactic gastrectomy. These protocols typically involve annual upper endoscopy with a comprehensive biopsy strategy, which includes obtaining a minimum of 30 non-targeted gastric mucosal biopsies from various anatomical regions of the stomach [64]. This approach aims to detect early-stage cancers or precancerous lesions, as the prognosis for symptomatic invasive diffuse carcinoma is exceedingly poor, with only about 10% of cases potentially curable at the time of diagnosis [48]. Studies have shown that endoscopic surveillance can identify occult SRCC in asymptomatic patients, with detection rates reported between 15% and 36% [65]. However, the overall efficacy of this surveillance strategy remains debated, as detection rates of early-stage cancer have been mixed, indicating that while surveillance is necessary, it may not be sufficient as a standalone strategy [66].

Moreover, the psychological and physical implications of delaying surgery must be considered. Many patients choose to postpone prophylactic gastrectomy due to concerns about surgical risks, recovery, and long-term health impacts, including nutritional deficiencies and changes in lifestyle. This decision underscores the importance of a well-structured surveillance program that not only monitors for cancer development but also provides psychological support and counseling to address the fears and anxieties associated with living with a high cancer risk [28].

Limitations of Surveillance

The limitations of surveillance methods in detecting early DGC, particularly in individuals with CDH1 mutations, are significant and multifaceted. One of the primary challenges is the submucosal spread of DGC, which often remains undetectable during endoscopic examinations. This characteristic of DGC is particularly problematic because the disease can progress without producing overt symptoms, leading to missed opportunities for early intervention. The microscopic nature of early-stage DGC, particularly the presence of SRCC, complicates detection further, as these lesions may not be visible during routine endoscopy [48].

Endoscopic surveillance typically relies on random biopsies to identify cancerous lesions. However, this method has been shown to have limited efficacy in detecting early-stage cancers, especially those that infiltrate deeper into the submucosa [67]. The random sampling approach can easily overlook small, localized lesions that have not yet caused significant mucosal changes, leading to a false sense of security for patients undergoing surveillance. Furthermore, the sensitivity of endoscopic techniques can vary significantly based on the experience of the endoscopist, which can further impact detection rates [52,64,67].

Another limitation is the inherent delay in the progression of gastric cancer from an early to an advanced stage. Studies indicate that early-stage gastric cancer can take several months to transition into a more advanced stage, during which time it may remain undetected [33]. This delay poses a significant risk, as patients may continue to live without awareness of their cancer status, potentially leading to a diagnosis at a much later stage, when treatment options are limited and prognosis is poor [68].

Moreover, the psychological implications of surveillance must not be overlooked. Patients may experience anxiety and uncertainty regarding their cancer risk, especially when surveillance results are inconclusive or when they are advised to continue with regular endoscopic evaluations without definitive findings [48]. This psychological burden can affect their QoL and may influence their decisions regarding prophylactic surgery or further surveillance.

Therefore, while endoscopic surveillance is a critical component of managing individuals with CDH1 mutations who decline prophylactic surgery, its limitations in detecting early DGC are pronounced. The submucosal nature of DGC, the reliance on random biopsies, the variability in endoscopist experience, and the psychological impact on patients all contribute to the challenges faced in early detection. These factors underscore the need for continued research into more effective surveillance strategies and the potential integration of advanced imaging techniques to enhance early cancer detection.

Molecular mechanisms and pathogenesis

Loss of E-cadherin Function

E-cadherin is essential for maintaining epithelial integrity and cell polarity through its function in adherens junctions. Its downregulation or loss is associated with several molecular pathways that contribute to tumorigenesis, invasion, and metastasis.

One of the primary consequences of E-cadherin loss is the disruption of cell-cell adhesion, which leads to increased cellular motility and the EMT [28]. During EMT, epithelial cells lose their characteristic features and acquire migratory and invasive properties, facilitating cancer spread. This transition is often regulated by transcription factors such as Snail and Slug, which repress E-cadherin expression and promote mesenchymal markers [69]. The loss of E-cadherin thus not only initiates EMT but also enhances the invasive potential of cancer cells, allowing them to breach the basement membrane and invade surrounding tissues [70].

Furthermore, E-cadherin functions as a tumor suppressor by inhibiting pathways that promote cell proliferation and survival. For instance, E-cadherin interacts with β-catenin, a key regulator of the Wnt signaling pathway. When E-cadherin is lost, β-catenin can translocate to the nucleus, where it activates transcription of genes that promote cell proliferation and survival, thereby contributing to tumor growth [71,72]. This aberrant activation of β-catenin signaling is particularly relevant in gastric cancer, where E-cadherin loss is frequently observed [73].

Additionally, the loss of E-cadherin affects cytoskeletal organization and signaling pathways involving Rho GTPases, which are crucial for maintaining cell shape and motility [24]. The cytoplasmic accumulation of p120-catenin, normally associated with E-cadherin, can lead to the activation of RhoA and Rac1 signaling pathways, promoting cell migration and invasion. This crosstalk between E-cadherin and integrins further complicates the cellular response to the extracellular matrix, enhancing the invasive behavior of cancer cells [37].

Moreover, E-cadherin loss is associated with the upregulation of various oncogenic pathways. For instance, the expression of integrin β1 is often increased in E-cadherin-deficient tumors, which correlates with enhanced invasive capabilities and poor prognosis [37]. The interplay between integrins and E-cadherin dysfunction highlights the complex signaling networks that drive cancer progression. Collectively, these changes facilitate tumor invasion and metastasis, underscoring the importance of E-cadherin as a key player in cancer biology.

Interaction With Environmental Factors

The interaction between CDH1 mutations and environmental factors, particularly diet and *H. pylori* infection, plays a significant role in the development of gastric cancer. CDH1, which encodes E-cadherin, is crucial for maintaining cell adhesion and tissue integrity. Mutations in this gene are associated with HDGC, where the penetrance of gastric cancer is notably high among mutation carriers, reaching nearly 100% in some studies [13,27]. However, environmental factors such as *H. pylori* infection can further influence the risk and progression of gastric cancer in individuals with CDH1 mutations.

*H. pylori* infection has been shown to induce epigenetic changes, including the hypermethylation of the CDH1 gene, which can lead to its silencing and contribute to gastric carcinogenesis [1,10]. For instance, a study indicated that patients with chronic gastritis infected by *H. pylori* exhibited higher levels of CDH1 methylation, suggesting that *H. pylori* may act as a promoter of CDH1 gene methylation during the early stages of gastric cancer development [40]. Additionally, the presence of inflammatory mediators associated with *H. pylori* infection can exacerbate the dysregulation of signaling pathways that are critical for cell proliferation and apoptosis, further compounding the risk for those with CDH1 mutations [41]. Moreover, dietary factors may also interact with CDH1 mutations and *H. pylori* infection to influence gastric cancer risk. Certain dietary patterns, particularly those high in salt and low in fruits and vegetables, have been implicated in gastric cancer development. These dietary habits may exacerbate the effects of *H. pylori* infection and the presence of CDH1 mutations, potentially leading to increased oxidative stress and inflammation in the gastric mucosa, which are known contributors to cancer development [74]. The interplay between genetic predisposition due to CDH1 mutations and environmental factors like *H. pylori* infection and diet highlights the multifaceted nature of gastric cancer etiology. Understanding these interactions is crucial for developing targeted prevention and treatment strategies for individuals at high risk due to genetic factors.

Emerging research and therapeutic approaches

Gene Therapy and Molecular Targeting

Emerging research on targeted therapies, gene editing, and drugs aimed at restoring E-cadherin function is gaining traction, particularly in the context of gastric cancer, where E-cadherin plays a critical role as a tumor suppressor. The loss of E-cadherin expression is associated with increased invasiveness and poor prognosis in gastric cancer patients [24]. Therefore, strategies to restore E-cadherin function or mimic its activity are being explored as potential therapeutic avenues.

One promising approach involves the use of small molecules to restore E-cadherin expression. For instance, the study by Jiang et al. demonstrated that pizotifen, a drug traditionally used for migraine treatment, can upregulate E-cadherin levels in gastric cancer cell lines, thereby inhibiting cell proliferation and invasion [75]. This suggests that repurposing existing drugs may provide a novel strategy for enhancing E-cadherin function in cancer therapy. Antibodies that can block the activity of proteins involved in E-cadherin degradation or inhibition are being developed. This approach can potentially prevent the loss of E-cadherin and restore its function [76].

Additionally, the use of gene editing technologies, such as CRISPR-Cas9, is being investigated to directly target and restore E-cadherin expression. A study by Herrera-Pariente et al. focused on the role of CTNND1, which interacts with E-cadherin, in early-onset gastric cancer. By employing CRISPR-Cas9 technology, the study aimed to elucidate the impact of CTNND1 variants on E-cadherin-mediated cell interactions, potentially paving the way for targeted genetic interventions [76,77]. This highlights the potential of gene editing to correct E-cadherin dysfunction at the genetic level.

Moreover, the identification of synthetic lethal interactions in E-cadherin-deficient cells has led to the exploration of targeted therapies that exploit these vulnerabilities. Bougen-Zhukov et al. reported that E-cadherin-deficient cells are sensitive to the multikinase inhibitor dasatinib, suggesting that targeting specific signaling pathways could provide therapeutic benefits for patients with gastric cancer characterized by low E-cadherin expression [78]. This approach underscores the importance of understanding the molecular context of E-cadherin loss to develop effective treatments. In addition to small molecules and gene editing, other strategies are being explored to enhance E-cadherin function. For example, research has indicated that inhibiting histone deacetylase 2 (HDAC2) can lead to increased E-cadherin expression, thereby reducing metastasis in gastric carcinoma [79]. This suggests that epigenetic modulation may also serve as a viable strategy for restoring E-cadherin function.

Overall, the restoration of E-cadherin function through various therapeutic strategies represents a promising area of research in gastric cancer treatment. By leveraging small molecules, gene editing technologies, and targeted therapies, there is potential to improve outcomes for patients with E-cadherin-related malignancies.

Immunotherapy and Targeted Treatments

The potential role of immunotherapy in CDH1-related gastric cancer, particularly HDGC, is an emerging area of research that is gaining attention due to the unique molecular characteristics associated with CDH1 mutations. CDH1 mutations lead to the loss of E-cadherin, a critical protein for cell adhesion, which is implicated in the aggressive nature of HDGC. This loss not only contributes to tumor progression but also influences the tumor microenvironment, making it a candidate for immunotherapeutic strategies.

One of the key molecular characteristics of CDH1-related gastric cancer is the high tumor mutation burden (TMB) observed in some cases. TMB is increasingly recognized as a biomarker for the efficacy of immune checkpoint inhibitors, such as PD-1 and PD-L1 blockers [80]. A study by Cai et al. highlighted that analyzing TMB through next-generation sequencing (NGS) can provide insights into the potential responsiveness of gastric tumors to immunotherapy [80]. Given that CDH1 mutations are associated with a distinct mutational landscape, understanding the TMB in these patients could help tailor immunotherapeutic approaches.

Moreover, the immune landscape in CDH1-related gastric cancer may differ from that in sporadic gastric cancers. The loss of E-cadherin can lead to an altered immune response, characterized by increased infiltration of immune cells, including T cells and macrophages, which may influence the effectiveness of immunotherapy [81]. Research indicates that the tumor microenvironment in E-cadherin-deficient tumors is often associated with a more immunosuppressive phenotype, which could hinder the efficacy of immune checkpoint inhibitors [70,81]. Therefore, strategies to enhance immune activation in this context are critical.

Additionally, the identification of specific biomarkers related to CDH1 mutations could further refine the application of immunotherapy. For instance, the study by Hu et al. emphasizes the importance of understanding the genetic and epigenetic alterations in HDGC to identify potential therapeutic targets and biomarkers for immunotherapy [71]. This could lead to the development of combination therapies that integrate immunotherapy with agents targeting the unique pathways activated by CDH1 loss. Furthermore, the exploration of novel therapeutic agents that can restore E-cadherin function or mimic its activity may also enhance the effectiveness of immunotherapy. For example, Bougen-Zhukov et al. discussed the potential of allosteric AKT inhibitors in targeting vulnerabilities in E-cadherin-deficient cells, which could synergize with immunotherapeutic approaches [82]. This highlights the need for a multifaceted approach that combines immunotherapy with strategies aimed at restoring E-cadherin function or modulating the tumor microenvironment.

Ethical and psychological considerations

Genetic counseling plays a crucial role for individuals with CDH1 mutations, particularly in the context of HDGC. The CDH1 gene encodes E-cadherin, a protein that is essential for cell adhesion, and mutations in this gene significantly increase the risk of developing gastric cancer and LBC [31]. Genetic counseling provides individuals and families with information about the implications of these mutations, including the risks of cancer, the options for surveillance, and the potential for prophylactic surgeries such as total gastrectomy, which is recommended for high-risk individuals [56]. The identification of CDH1 mutations can occur through various genetic testing methods, including multigene panel testing, which has revealed pathogenic mutations even in individuals without a clear family history of gastric cancer [31]. This underscores the importance of genetic counseling in interpreting test results and guiding decisions about family planning and cancer prevention strategies.

Family planning considerations are particularly complex for those with CDH1 mutations. Genetic counseling can help prospective parents understand the inheritance patterns of CDH1 mutations, which follow an autosomal dominant pattern, meaning that each child has a 50% chance of inheriting the mutation [38]. Furthermore, the psychosocial impact of knowing one carries a CDH1 mutation can be significant. Individuals may experience anxiety regarding their own health and the health of their children, as well as the emotional burden associated with the decision to undergo prophylactic surgery [63]. Support services, including psychological counseling and support groups, can provide essential resources for coping with these challenges, helping individuals navigate the emotional landscape that accompanies genetic testing and the potential for life-altering surgical decisions [63].

The psychological burden associated with genetic testing and the decision-making process regarding prophylactic surgery cannot be understated. Many individuals report feelings of uncertainty and fear about their cancer risk, which can be exacerbated by a lack of familial context if they are the first in their family to be tested. The role of support services is vital in addressing these concerns, offering not only emotional support but also practical guidance on managing health risks and making informed choices about surveillance and preventive measures. Additionally, the psychosocial implications of living with a CDH1 mutation extend beyond the individual to affect family dynamics and relationships, making comprehensive support an essential component of genetic counseling for mutation carriers [63].

Current guidelines and clinical recommendations

The management of individuals carrying pathogenic CDH1 mutations, particularly in the context of HDGC, is guided by a set of clinical recommendations that emphasize early detection and preventive measures. The IGCLC has established protocols that advocate for PTG as the primary intervention for CDH1 mutation carriers, especially given the high risk of developing gastric cancer at a young age. Studies indicate that nearly 100% of asymptomatic CDH1 carriers undergoing PTG have cancer foci identified in their gastric tissue, underscoring the silent nature of the disease and the necessity of surgical intervention [66].

For those who opt against prophylactic surgery, rigorous endoscopic surveillance is recommended. However, the effectiveness of endoscopic surveillance is limited due to the focal nature of HDGC, with detection rates for SRCC being notably low, often around 45% to 50% [44]. The IGCLC guidelines suggest that all visible lesions during endoscopy should be biopsied, along with random biopsies from multiple gastric regions to maximize the chances of detecting malignancy. Despite these measures, the consensus remains that PTG is the superior approach for managing CDH1 mutation carriers due to the high likelihood of undetected cancer [50].

In addition to gastric cancer risks, female CDH1 mutation carriers face a significant risk of developing LBC. Consequently, annual breast surveillance via MRI is recommended starting at age 30, with consideration for prophylactic mastectomy in cases with a strong family history of breast cancer [83]. This dual surveillance strategy reflects the multifaceted cancer risks associated with CDH1 mutations and aims to provide comprehensive management for affected individuals. Overall, the clinical guidelines for CDH1 mutation carriers emphasize a proactive approach, advocating for prophylactic gastrectomy as the gold standard while recognizing the need for alternative surveillance strategies for those who decline surgery. The integration of genetic counseling and multidisciplinary care is essential in managing the complex needs of these patients.

Expert Consensus

The clinical management of HDGC has evolved significantly, with expert consensus emphasizing the importance of genetic counseling, surveillance, and prophylactic surgical interventions. The IGCLC has established guidelines for the identification of families at risk for HDGC. These guidelines recommend genetic testing for individuals with a family history of gastric cancer, particularly if there are two or more documented cases of DGC in first- or second-degree relatives, with at least one diagnosed before the age of 50 [27]. The identification of CDH1 mutations is crucial, as it directly influences management strategies, including the consideration of PTG, which is the most effective method to reduce the risk of developing gastric cancer in mutation carriers [48].

Surveillance strategies for individuals with identified CDH1 mutations typically involve regular upper gastrointestinal endoscopies (EGD) starting at an early age, although the efficacy of such surveillance remains debated due to the often normal findings in patients who later develop cancer [43]. The consensus is that, while endoscopic surveillance may not be sufficient alone, it can be part of a comprehensive management plan that includes consideration of prophylactic surgery [48]. Furthermore, the management of HDGC should also consider the associated risks of LBC, particularly in women, where the lifetime risk is estimated at 42% [29,83]. This necessitates a multidisciplinary approach involving genetic counseling, oncological assessment, and surgical planning, tailored to the individual's family history and genetic findings [43].

## Conclusions

HDGC is a hereditary syndrome predominantly caused by CDH1 gene mutations, leading to a significant increase in the risk of developing diffuse-type gastric cancer. This aggressive cancer presents unique challenges for early detection and management due to its infiltrative nature and poor prognosis. Understanding the genetic basis of HDGC is essential for improving risk assessment and implementing effective surveillance strategies. Continued research into the molecular pathways affected by CDH1 loss may pave the way for targeted therapies, enhancing the clinical management of affected individuals and their families. Ultimately, increased awareness and genetic testing can contribute to better outcomes and informed decision-making for those at risk.
